# Dual-Spectral Plasmon-Induced Transparent Terahertz Metamaterial with Independently Tunable Amplitude and Frequency

**DOI:** 10.3390/nano11112876

**Published:** 2021-10-28

**Authors:** Tong Wu, Guan Wang, Yang Jia, Yabin Shao, Chen Chen, Jing Han, Yang Gao, Yachen Gao

**Affiliations:** 1 Electronic Engineering College, Heilongjiang University, Harbin 150080, China; 124wutong@163.com (T.W.); wang2687220886@163.com (G.W.); jiayang_1990@163.com (Y.J.); hanjing1980@163.com (J.H.); 2 Department of Computer & Electrical Engineering, East University of Heilongjiang, Harbin 150086, China; shao_yabin@163.com (Y.S.); rukawa1600@163.com (C.C.)

**Keywords:** plasmon-induced transparency, terahertz, graphene, strontium titanate, slow light

## Abstract

A bifunctional tunable metamaterial composed of pattern metal structure, graphene, and strontium titanate (STO) film is proposed and studied numerically and theoretically. The dual plasmon-induced transparency (PIT) window is obtained by coupling the bright state cut wire (CW) and two pairs of dark state dual symmetric semiring resonators (DSSRs) with different parameters. Correspondingly, slow light effect can also be realized. When shifting independently, the Fermi level of the graphene strips, the amplitudes of the two PIT transparency windows and slow light effect can be tuned, respectively. In addition, when independently tuning the temperature of the metamaterial, the frequency of the dual PIT windows and slow light effect can be tuned. The physical mechanism of the dual-PIT was analyzed theoretically by using a three-harmonic oscillator model. The results show that the regulation function of the PIT peak results from the change of the oscillation damping at the dark state DSSRs by tuning conductivity of graphene. Our design presents a new structure to realize the bifunctional optical switch and slow light.

## 1. Introduction

Electromagnetically induced transparency (EIT) is an effect resulting from quantum destructive interference. It can generate a narrow-band transparent window when light propagates through an originally opaque medium [1,2]. However, its application is limited due to harsh production conditions of the stable optical pumping and low temperature. Compared with the traditional EIT effect, plasmon-induced transparency (PIT) effect overcomes these harsh conditions [3]. Recently, many researchers have focused on various metamaterial structures to achieve PIT, which is the analog of EIT effect [4,5,6,7,8]. Previous studies have shown that PIT effect can be achieved mainly via bright and bright mode, bright and dark mode, and bright and quasi-dark mode [9,10,11,12]. At a PIT peak region, strong dispersion can occur, causing slow light effect which can be used in optical information processing [13,14,15,16].

In order to meet various practical applications, the properties of PIT should be able to be tuned. The tuning can be realized by changing structural parameters, using tunable materials and microelectromechanical systems (MEMS) technology. Due to the high flexibility, tunable materials-based PIT devices have become a research hotspot [17,18,19,20,21,22,23,24]. Graphene is especially widely used in the design of tunable PIT devices because of its high electron mobility, high modulation depth, tunable surface conductivity and low insertion loss characteristics. Tunable graphene-based PIT devices can realize different functions, such as the single-PIT [18,19,20], dual-PIT [21,22,23] and multi-PIT [24]. Recently, STO has also aroused the interest of researchers due to its temperature-tunable relative permittivity [25]. In 2020, Zhong proposed a tunable PIT metamaterial based on STO. When the device temperature is changed, the frequency of the single PIT window and slow light effect can be adjusted [26]. However, we can see most of the work mentioned above focused mainly on the tuning of either amplitude or frequency of the PIT peak. For example, in references [18,19,22,23], researchers proposed the metamaterials to realize the regulation of the PIT peak intensity. In references [20,21,24,26], researchers realized the regulation of the frequency of the PIT peak. To our knowledge, the tunable PIT metamaterial with independently tunable amplitude and frequency of dual-PIT effect has not been previously reported. 

In this paper, we designed a metal structure to achieve the dual PIT effect and realized the modulation of the intensity and frequency of the PIT effect by changing the Fermi level of graphene strips and the temperature of STO films. The mechanisms of the tunable dual-PIT effect were analyzed using a three-harmonic oscillator model.

## 2. Materials and Methods

**Figure 1:**
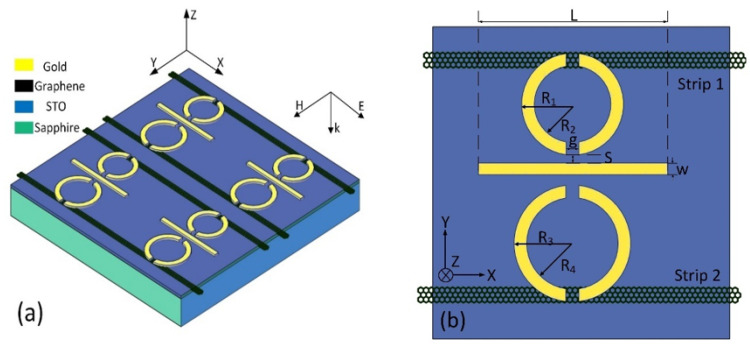
(**a**). Three-dimensional diagram of the hybrid metamaterial. (**b**) The top view of the unit cell.

The PIT metamaterial proposed is illustrated in Figure 1. From Figure 1a, it can be observed that the metamaterial is composed of three layers, which are graphene–metal structure, STO film, and sapphire substrate. The parameters of metamaterial are shown in Figure 1b. Two sets of symmetrical half-rings named upper double symmetric semiring resonators (UDSSRs) and bottom double symmetric semiring resonators (BDSSRs) are placed on the upper and bottom sides of cut wire (CW), respectively. The thickness of the metal structure is 0.2 μm. The length of CW is *L* = 80 μm, and the width is w = 5 μm. For UDSSRs and BDSSRs, the gap width of splits is g = 5 μm. The outer radius and inner radius of UDSSR are 20 μm and 15 μm, respectively. The outer radius and inner radius of BDSSRs are 23 μm and 18 μm, respectively. The distance between two DSSRs and CW is *S* = 3.5 μm. Under the UDSSRs and BDSSRs, there are two graphene strips defined as strip 1 and strip 2, respectively. The STO film with a thickness of 100 nm is placed between the metal–graphene hybrid structure and sapphire substrate. The period of metamaterial is *P_x_* = *P_y_* = 120 μm.

Finite-difference time-domain (FDTD) algorithm is used for numerical simulation and calculating the near-field coupling of this design. The boundary conditions of x and y direction are periodic boundaries, and a perfect matching layer is used in the z direction. The x-polarized terahertz wave is incident vertically along the z direction. In the low frequency terahertz band, as a loss metal, gold can be represented by a static model, and its conductivity is 4.56 × 10^7^ S/m [27]. The refractive index of the sapphire substrate is 1.78 [28].

As a 2D material, the electric-magnetic properties of graphene can be described by surface conductivity $\sigma_{g}$. According to random-phase approximation (RPA) theory, the conductivity $\sigma_{g}$ can be expressed by combining intraband $\sigma_{intra}$ and interband $\sigma_{inter}$ [29]:

(1) $$\sigma_{g}=\sigma_{intra}+\sigma_{inter}\;=\frac{2e^{2}k_{B}T}{\pi \hbar^{2}}\frac{i}{\omega +i\tau^{-1}}ln\left[2cosh\left(\frac{E_{F}}{2k_{B}T}\right)\right]\;+\frac{e^{2}}{4\hbar }\left[\frac{1}{2}+\frac{1}{\pi }arctan\left(\frac{\hbar \omega -2E_{F}}{2k_{B}T}\right)\;-\frac{i}{2\pi }ln\frac{{\left(\hbar \omega +2E_{F}\right)}^{2}}{{\left(\hbar \omega -2E_{F}\right)}^{2}+4{\left(k_{B}T\right)}^{2}}\right]$$

where e is the electron charge, $k_{B}$ is the Boltzmann constant, T is the temperature in Kelvin, ℏ is the reduced Planck’s constant, ω is the THz frequency, τ and $E_{F}$ are graphene carrier relaxation time and Fermi level. 

For STO materials, the temperature-dependent relative permittivity in the THz spectral region is [30]:

(2) $$\varepsilon_{\omega }=\varepsilon_{\infty }+\frac{f}{\omega_{0}-\omega^{2}-i\omega \gamma }$$

where $\varepsilon_{\infty }$ is 9.6, representing the high-frequency bulk permittivity, f is an oscillator strength depending on temperature, with a value of $2.36\times 10^{6}{\;\mathrm{cm}}^{2}$ [31]. ω, $\omega_{0}$ and γ are the angular frequency, soft mode frequency and the damping factor, respectively. The formula related to temperature can be expressed as:

(3) $$\omega_{0}\left(T\right)\;\left[{\mathrm{cm}}^{-1}\right]=\sqrt{31.2\left(T-42.5\right)}$$

(4) $$\gamma \left(T\right)\;\left[{\mathrm{cm}}^{-1}\right]=-3.3+0.094T$$

where T is temperature of STO. $\omega_{0}\left(T\right)$ can be obtained using the Cochran law and γ(T) can be fitted by an empirical linear dependence. We can see from Equations (2)–(4) that the relative permittivity of STO under different angular frequency and temperature can be calculated.

## 3. Results and Discussions

**Figure 2:**
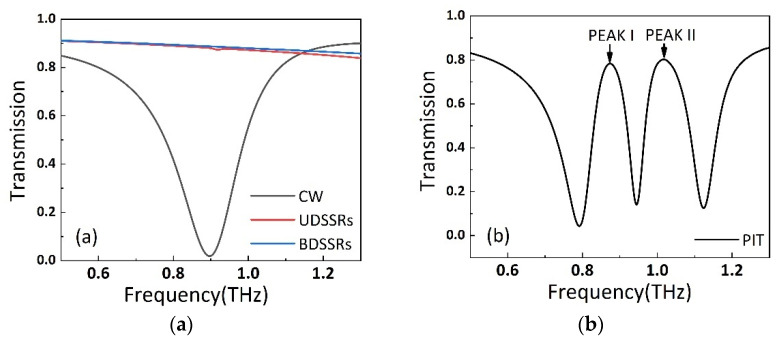
(**a**) The transmission spectra of isolated CW array, BDSSRs array and UDSSRs array. (**b**) The transmission spectra of the PIT metamaterial composed of two parts of DSSRs and CW.

In order to investigate the mechanism of double PIT transparency windows, we conducted the simulation for four arrays, composed of CW arrays, UDSSRs, BDSSRs and a combined array of them. Here, the temperature of STO was set to be 425 K. In Figure 2a, it can be seen that when the x-polarization plane wave achieves a coupling with the single CW structure, a transmission valley appears at 0.9 THz because of the localized surface plasmon resonance (LSPR) at CW. However, due to the symmetry of the isolated DSSRs structure with respect to the x-polarization incident field, the UDSSRs or BDSSRs are inactive at the same frequency [32]. Thus, the CW and the two pairs of DSSRs behave as bright and dark resonance modes, respectively. When the CW, UDSSRs and BDSSRs are combined into a unit cell, under x-polarized electric field excitation, two PIT windows arise because of the destructive interference caused by the coupling of the two LC resonance modes and LSPR mode. As shown in Figure 2b, two transparent windows at 0.87 THz and 1.016 THz can be observed and denoted as peak I and peak II.

**Figure 3:**
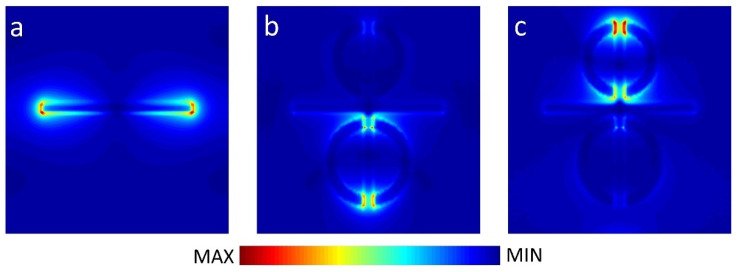
Electric field distributions of (**a**) the CW, (**b**) the PIT metamaterials at 0.873 THz, and (**c**) the PIT metamaterials at 1.016 THz.

**Figure 4:**
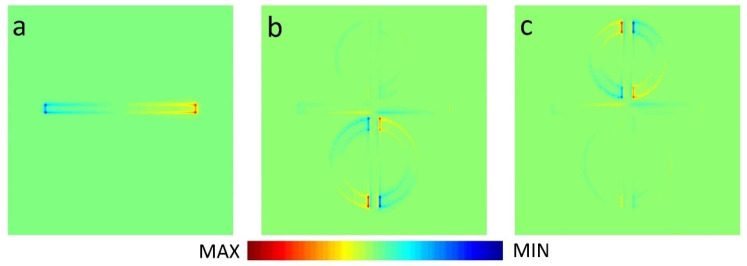
Charge distribution of (**a**) the CW, (**b**) the PIT metamaterials at 0.873 THz, and (**c**) the PIT metamaterials at 1.016 THz.

In order to analyze the mechanism of PIT effect, we studied the electric field and charge distribution at resonance frequency. As shown in Figure 3a and Figure 4a, when CW is coupled with a plane wave, it can be observed that there is a strong electric field at the *x*-axis edges and corners of CW, and the charges are concentrated in the same position. This phenomenon, which can be excited, directly belongs to LSPR and can be described as bright resonance mode. When BDSSRs, UDSSRs and CW are placed in the arrays to achieve coupling, the electric field in 0.873 THz and 1.016 THz are shown in Figure 3b,c, the charge distribution is shown in Figure 4b,c. 

From Figure 3b and Figure 4b, we can see that the enhancement of the electric field and accumulation of opposite charge transfer from the edges and corners of CW to the splits of BDSSRs. Similarly, in Figure 3c and Figure 4c, we can see the electric field enhancement and opposite charge transfer to the splits of UDSSRs. These two resonance modes generated by indirect coupling with CW belong to the LC resonance and can be regarded as dark modes. Due to the phase difference of π between bright resonance mode and dark resonance mode, destructive interference will occur between LSPR and LC resonance, which results in the appearance of transparent windows [33].

**Figure 5:**
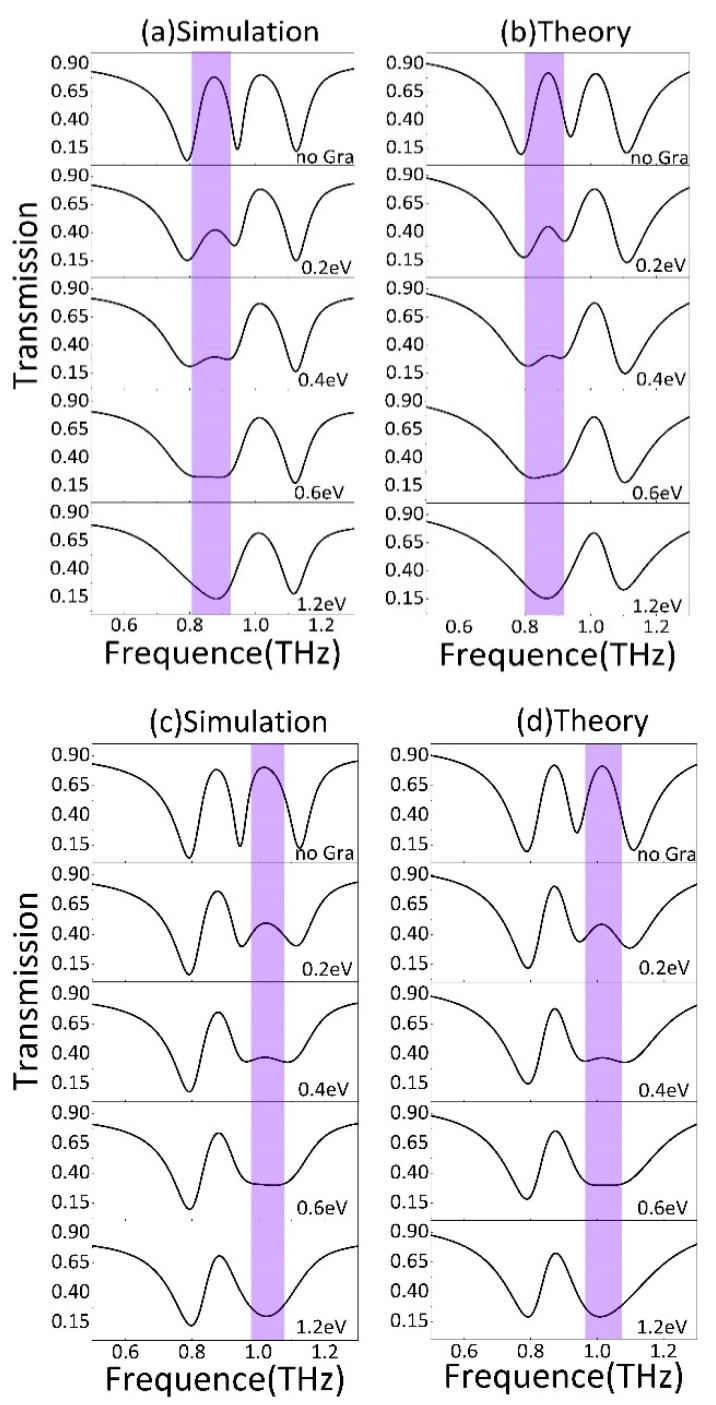
(**a**) The simulated and (**b**) analytical fitted transmission spectrum with different Fermi levels of strip 2. (**c**) The simulated and (**d**) analytical fitted transmission spectrum with different Fermi levels of strip 1.

Next, the individually tunable properties of the device are analyzed. Figure 5 shows the simulated and theoretical transmission spectrum with different Fermi levels of strip 2 and strip 1, respectively. In Figure 5a,c, it can be found that the two PIT transparency windows of this metamaterial can be achieved, and the independent on-to-off switching function at two PIT windows can be realized by tuning the graphene Fermi level. Figure 5a (top panel) is the transmission spectra when the graphene strips are absent. The amplitude of transmission of peak I and peak II are 0.7814 and 0.8017, respectively. When strip 2 is placed under the splits of the BDSSRs and the Fermi level is set to 0.2 eV, the transmission of peak I reduces to 0.424. As the graphene Fermi level increases, peak I undergoes a continuous decrease, whereas peak II changes minimally. Previous studies have shown that the graphene Fermi level can be modulated to be 1.2 eV [34]. When the Fermi level increases to 1.2 eV, peak I disappears completely, which causes an off state. In order to quantitatively describe the modulation depth of the PIT transparent windows, we introduce the formula $\Delta T=\left[\left(T_{0}-T_{g}\right)/T_{0}\right]\times 100\%$, where $T_{0}$ and $T_{g}$ refer to the amplitude of transmission peak without and with graphene, respectively. Finally, with the Fermi level of 1.2 eV, the transmission of peak I reduces to 0.137, correspondingly the modulation depth of peak I is calculated to be 82.4% using the formula.

In Figure 5c, it can be observed that, as the Fermi level of strip 1 increases from 0.2 eV to 1.2 eV, the transmission change of peak II is similar to that of peak I; namely, the amplitude of peak II decreases with the increase in the graphene Fermi level. When the graphene Fermi level reaches1.2 eV, the transmission of peak II is 0.2022. The modulation depth of peak II can achieve 74.7%. Therefore, this design can realize the optical switch-like regulation of peak I and peak II by adjusting the Fermi level of strip 1 and strip 2, respectively. 

In order to further investigate the independent tunable mechanism of the dual-PIT transparency window by tuning the graphene Fermi level, we analyzed the interaction of the bright and two dark modes using the three-harmonic oscillator model [35]. As a bright mode, the LSPR at CW can be represented by oscillator 1 arising from direct coupling with the plane wave. As the dark modes excited through near field coupling with the bright mode, the BDSSRs and UDSSRs are represented by oscillator 2 and 3, respectively. The coupling effect between the three resonance modes is described by the following formula:

(5) $${\ddot{x}}_{0}\left(t\right)+\gamma_{0}{\dot{x}}_{0}\left(t\right)+\omega_{0}^{2}x_{0}\left(t\right)+\kappa_{1}{\dot{x}}_{1}\left(t\right)+\kappa_{2}{\dot{x}}_{2}\left(t\right)=\lambda_{0}E$$

(6) $${\ddot{x}}_{1}\left(t\right)+\gamma_{1}{\dot{x}}_{1}\left(t\right)+\omega_{1}^{2}x_{1}\left(t\right)-\kappa_{1}{\dot{x}}_{0}\left(t\right)=0$$

(7) $${\ddot{x}}_{2}\left(t\right)+\gamma_{2}{\dot{x}}_{2}\left(t\right)+\omega_{2}^{2}x_{2}\left(t\right)-\kappa_{2}{\dot{x}}_{0}\left(t\right)=0$$

Here, *E* represents the incident electromagnetic field, $\lambda_{0}$ describes the coupling strength of the electromagnetic field. $\omega_{0},\;\omega_{1},$ $\omega_{2}$ are the resonance frequencies of oscillator 1, oscillator 2 and oscillator 3, respectively. $x_{0}$ and $\gamma_{0}$ are the amplitude and damping of the bright resonance mode.$\;x_{1}$ and $x_{2}$ are the amplitudes of the dark resonance mode at BDSSRs and UDSSRs, respectively, and $\gamma_{1}$ and $\gamma_{2}$ are the damping of the dark resonance mode at BDSSRs and UDSSRs, respectively. The coupling coefficients between the two dark state modes and the bright state are $\kappa_{1}$ and $\kappa_{2}$, respectively. After solving the Equations (5)–(7) with $\omega^{2}-{\omega_{b}}^{2}\approx \left(\omega -\omega_{b}\right)\cdot 2\omega$, and $\lambda =\lambda_{0}/2\omega$, the susceptibility χ of the PIT metamaterials can be obtained as:

(8) $$\chi \left(\omega \right)=(\chi_{r}+i\chi_{i})\propto \frac{1}{A}\left(\omega_{2}-\omega -i\frac{\gamma_{1}}{2}\right)\left(\omega_{3}-\omega -i\frac{\gamma_{2}}{2}\right)$$

where:

(9) $$A=\left(\omega_{2}-\omega -i\frac{\gamma_{1}}{2}\right)\left(\omega_{1}-\omega -i\frac{\gamma_{0}}{2}\right)\left(\omega_{3}-\omega -i\frac{\gamma_{2}}{2}\right)-\frac{\kappa_{1}^{2}}{4}\left(\omega_{3}-\omega -i\frac{\gamma_{2}}{2}\right)\;-\frac{\kappa_{2}^{2}}{4}\left(\omega_{2}-\omega -i\frac{\gamma_{1}}{2}\right)$$

In Equation (8)$\;\chi_{r}$ represents the dispersion. The transmittance T can be calculated by the formula $T=1-\lambda_{0}\chi_{i}$, where $\chi_{i}$ is proportional to the energy loss [17,36].

**Figure 6:**
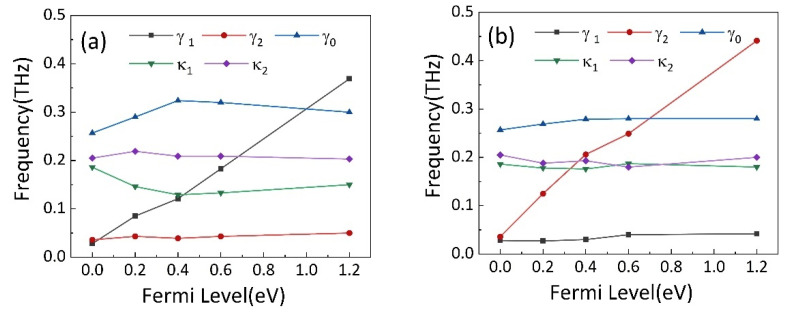
The variations of $\gamma_{1}$, $\gamma_{2}$, $\gamma_{0}$, $\kappa_{1}$ and $\kappa_{2}$ with different Fermi levels of (**a**) strip 2 and (**b**) strip 1.

Figure 5b,d show the theoretical results of the transmission spectrum. It is observable that they are in strong agreement with the simulation results shown in Figure 5a,c. Correspondingly, the fitting parameters are obtained and shown in Figure 6a,b. In Figure 6a, it can be found that the damping rate of the dark mode $\gamma_{1}$ has a significant increase from 0.025 THz for the case of no graphene to 0.65 THz for the case of Fermi level of 1.2 eV, whereas the fitting parameters $\gamma_{2}$, κ, and δ remain roughly unchanged. This phenomenon indicates that the increased Fermi level of strip 2 leads to an increased damping $\gamma_{1}$ at BDSSRs. In this design, as the Fermi level increases, the conductivity of the graphene strip connecting the two SSRs increases. When the Fermi level is 1.2 eV, the LC resonance at BDSSRs is hindered. Consequently, the destructive interference between BDSSRs and CW is weakened and peak I disappears. 

On the other hand, when the Fermi level of strip 1 is changed from 0.2 eV to 1.2 eV, in Figure 6b, we can see the fitting parameters $\gamma_{1}$, κ and δ remain basically unchanged, whereas the damping rate $\gamma_{2}$ of dark mode increases significantly from 0.025 THz to 0.6 THz with the changing of Fermi level from 0.2 eV to 1.2 eV. This phenomenon can be explained by a similar principle; namely, as the Fermi level of increases, the increase in the conductivity of strip 1 reduces the intensity of LC resonance caused by the coupling of UDSSRs and CW, resulting in the weakening of destructive interference. The increase in damping rate $\gamma_{2}$ eventually leads to a disappearance in peak II.

**Figure 7:**
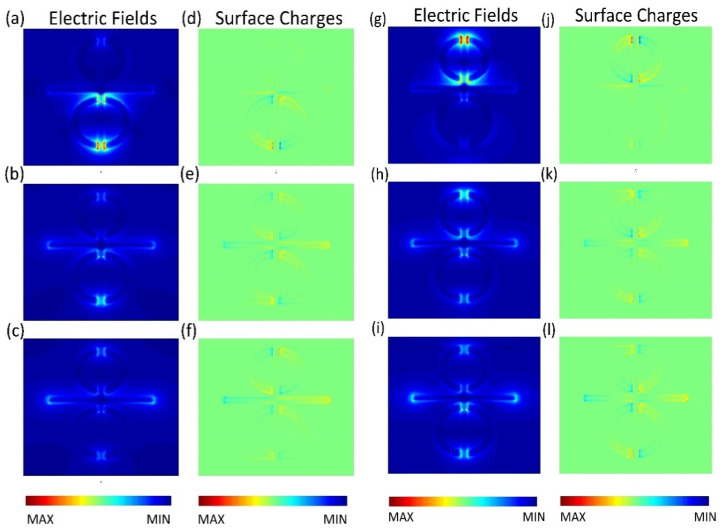
The distribution of (**a**–**c**) electric field and (**d**–**f**) surface charge at the PIT resonance with different strip 2 Fermi levels of No Gra, 0.4 eV and 1.2 eV. The distribution of (**g**–**i**) electric field and (**j**–**l**) surface charge at the PIT resonance with different strip 1 Fermi level of No Gra, 0.4 eV and 1.2 eV.

In order to further explain the physical mechanism of the tunable metamaterials, in Figure 7, we present the distributions of the electric field and charge at resonance peak I and peak II. The electric field and charge distributions at peak I with different Fermi levels of strip 2 are shown in Figure 7a–f. In the absence of strip 2, as shown in Figure 7a,d, a strong electric field and accumulation of opposite charges are observed at the splits of BDSSRs. Thus, the dark mode at BDSSRs provides weak damping. When placing strip 2 under the BDSSRs and changing the Fermi level to 0.4 eV, the distribution of the electric field and charge are shown in Figure 7b,e. It is obvious that the electric field and opposite charge distribution at the splits decreases, while the electric field at CW and the opposite charge increases. This is due to the fact that the charges that accumulated opposingly at the splits are neutralized by the conductive graphene, which causes the weakening of the LC resonance at BDSSR, resulting in the weakening of the destructive interference between LC resonance at BDSSR and LSPR at CW. When the graphene Fermi level is 1.2 eV, the simulation result is shown in Figure 7c,f. We can see the electric field enhancement and the opposite charge accumulation at BDSSRs almost disappear. However, the electric field enhancement at CW is recovered and the opposite charges are re-accumulated on both edges and corners of CW. The reason is that the graphene strip almost completely neutralizes the opposite charges at the BDSSRs splits caused by the strong recombination effect of the monolayer graphene and the damping of the dark BDSSRs is too large to support LC resonance. 

Therefore, it can be concluded that the damping enhancement caused by the increase in the graphene Fermi level weakens the LC resonance mode at BDSSRs and the LSPR mode, which disappears due to the destructive interference between CW and BDSSRs recovering gradually.

Figure 7g–l show the distributions of the electric field and charge at peak II with different Fermi levels of strip 1. In Figure 7g,j, when the graphene strip is not implanted into UDSSRs, a strong electric field and opposite charges are concentrated at the splits and the dark UDSSRs exhibit a low damping when placing strip 1 under the UDSSRs and changing the Fermi level to be 0.4 eV. From Figure 7h,k, it can be found that the intensity of the electric field at the UDSSRs splits, becomes weaker, and the charge density decreases, whereas the electric field intensity and charge density of CW increase gradually. With the maximum Fermi level of 1.2 eV, the simulation result is shown in Figure 7i,l, the opposite charges at UDSSRs are almost completely neutralized and the intensity of the electric field almost disappears, resulting in the disappearance of LC resonance. The intensity of the electric field and the opposite charge at CW are further recovered to achieve a strong LSPR. This is due to the fact that the high Fermi level of graphene causes the damping at UDSSRs to be too large to support the LC resonance modes, and leads to a disappearance of the destructive interference between the UDSSRs and CW. Therefore, the origin of the independent modulation of two PIT resonance can be attributed to the actively tunable Fermi level of the graphene strips under the BDSSRs and UDSSRs. 

**Figure 8:**
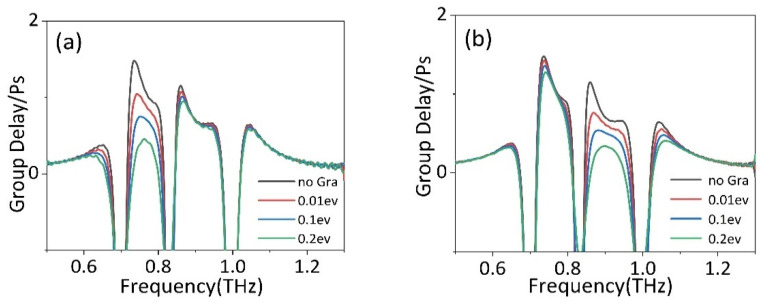
The group delay of PIT metamaterial with different Fermi level of (**a**) strip 2 and (**b**) strip 1.

Previous studies have shown that the PIT effect is usually accompanied by the changing of dispersion properties and causes the light to slow down. Generally, group delay can be used to describe the slow light effect quantitatively [37], which can be described as:

(10) $$t_{g}=\frac{d\varphi }{d\omega }$$

where φ and ω=2πf are the transmission phase shift and frequency. The temperature of STO in this part is maintained at 425 K. Figure 8a,b shows the group delay of devices with different Fermi levels of strip 2 and strip 1, and it is evident that two parts of group delay achieve a good modulation. In the absence of graphene, the phase produces a steep jump at two transparent windows generated by PIT effect, resulting in the group delay of 1.47 ps and 1.15 ps. When strip 2 is placed under BDSSRs, the group delay change of Fermi level from 0.01 eV to 0.2 eV is shown in Figure 8a. It can be found that with the increase in Fermi level, the group delay generated at peak I gradually decreases. Finally, when the Fermi level is 0.2 eV, the group delay at peak II is only 0.45 ps. However, the group delay at peak II decreases slightly and still maintains the group delay of 0.98 ps.

Similarly, as shown in Figure 8b, the group delay at peak II decreases gradually by increasing the Fermi levels of strip 1 from 0.01 eV to 0.2 eV. When the Fermi level increases to 0.2 eV, the group delay at peak II gradually decreases to 0.32 ps. However, the group delay at peak I also decreases slightly and still maintains the group delay of 1.27 ps. Therefore, this design can modulate two slow light effects independently and continuously by shifting the graphene Fermi level, which is of great research significance for devices with independent tunable dual slow light.

**Figure 9:**
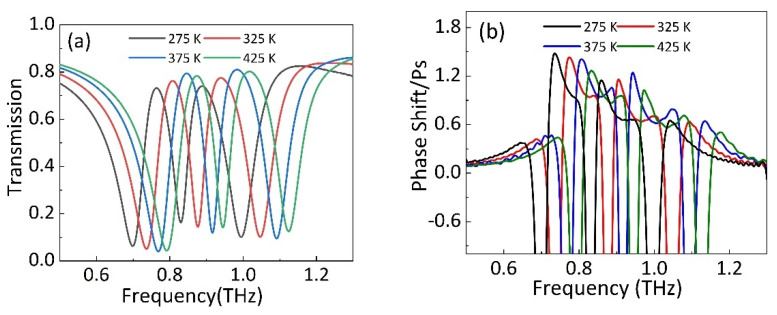
In the absence of graphene strips, (**a**) the transmission and (**b**) group delay of PIT metamaterial with different temperature of STO film.

In this section, we discuss the influence of temperature on the PIT effect. In the absence of strip 1 and strip 2, by modulating the temperature of STO, the changing of transmission spectrum is shown in Figure 9a. It can be found that peak I and peak II show blue shift. Specifically, as the temperature increases from 275 K to 425 K, the frequency of peak I moves from 0.76 THz to 0.87 THz, and the frequency of peak II moves from 0.88 THz to 1.01 THz. 

Figure 9b shows the frequency change of group delay by tuning the STO temperature. When the temperature of STO film increases, the two parts of group delay caused by double PIT effect can achieve blue shift with increasing temperature. Specifically, as the temperature increases from 275 K to 425 K, the peak frequency of the two group delay moves from 0.73 THz to 0.83 THz and 0.85 THz to 0.97 THz, respectively. Therefore, the frequency selection function of double PIT windows and group delay can be realized by tuning the temperature of STO film. 

Since the frequency of the PIT peak is affected by the LC resonance produced by the dark mode DSSRs, the DSSRs can be regarded as a frequent-selective surface and the resonance frequency can be estimated by [38,39]:

(11) $$f=\frac{c}{2\pi R\sqrt{\varepsilon_{eff}}}$$

where R is the effective radius of the DSSRs, and R is constant because of the fixed parameters of DSSRs. $\varepsilon_{eff}$ and c are effective dielectric constants of STO and the light speed in free space, respectively. In simulation, the $\varepsilon_{eff}$ decreases with the temperature increasing, so the frequencies of PIT peak and group delays both cause the blue shift. Therefore, this design can not only realize the amplitude tuning of the PIT transparency windows and the slow light effect, but also realize the selection of the resonance frequency of the dual PIT transparency windows and the slow light effect.

## 4. Conclusions

In conclusion, we achieved the modulation of double PIT effect by integrating monolayer graphene strips and STO film into PIT metamaterials. The simulation results show that the two PIT peaks can realize the on-to-off modulation by independently shifting the Fermi level of strip 1 and strip 2. The coupling effect in the PIT metamaterial has been studied using the three-harmonic oscillator model, and the theoretical analysis shows that the recombination effect of the conductive graphene will cause the changing of dark mode damping, resulting in the tuning of the PIT peak amplitude. Through the study of the slow light effect of this metamaterial, it was found that two group delays of this metamaterial can be tuned independently. In addition, the frequency selection function of the double PIT transparency window and the double slow light is also realized by controlling the temperature of the STO layer. The multi-functional controllable metamaterial realizes the amplitude control and frequency selection of PIT transparency windows and provides a new path for future PIT control and slow light tuning devices.

## Data Availability

All content and data have been displayed in the manuscript.
